# Proton Pump Inhibitors and Risk of Chronic Kidney Disease: A Systematic Review and Meta-Analysis

**DOI:** 10.7759/cureus.102220

**Published:** 2026-01-24

**Authors:** Bandar A Almabruk, Ghadah A Alsulami, Atheer Alzahrani, Eman A Alsulami, Abdulrahman A Badawood, Ahad S Alghanim, Fatimah F Alresheedi, Raghad S Alharbi, Hisham A Alghamdi

**Affiliations:** 1 Internal Medicine, King Salman Hospital, Riyadh, SAU; 2 Medical Laboratory Technology, University of Hail, Hail, SAU; 3 Medicine, King Saud bin Abdulaziz University for Health Sciences, Jeddah, SAU; 4 Medicine, King Abdulaziz University Faculty of Medicine, Jeddah, SAU; 5 Surgery, Jubail General Hospital, Jubail, SAU; 6 Pharmacy, Qassim University, Buraydah, SAU; 7 Medicine and Surgery, Al Baha University, Al Baha, SAU

**Keywords:** chronic kidney disease, ckd progression, end-stage renal disease, proton pump inhibitors, renal outcomes

## Abstract

Proton pump inhibitors (PPIs) are widely prescribed for acid-related disorders, yet growing evidence suggests a potential association between long-term PPI use and chronic kidney disease (CKD). This systematic review and meta-analysis aimed to evaluate the risk of CKD, disease progression, and end-stage renal disease (ESRD) among PPI users compared with nonusers or histamine-2 receptor antagonist users. A comprehensive search identified observational studies assessing renal outcomes among adult PPI users. Fifteen studies met the inclusion criteria, representing diverse populations across Asia, Europe, and the United States. Data on CKD incidence, progression, ESRD, and additional adverse effects were extracted. Pooled effect estimates were calculated using random-effects models. Heterogeneity, sensitivity analyses, and publication bias were evaluated using I², leave-one-out testing, and funnel plots. Our review included 15 studies, with sample sizes ranging from 3,023 to 462,421 participants. Meta-analysis of six studies (n = 594,680) demonstrated a significantly increased risk of incident CKD among PPI users (RR = 1.68, 95% CI: 1.20-2.34; p = 0.002; I² = 99%). Two studies (n = 171,583) assessing CKD progression showed a higher, but statistically nonsignificant, risk with PPI use (RR = 1.49, 95% CI: 0.84-2.65; p = 0.17; I² = 99.3%). Four studies (n = 149,702) examining ESRD found a modest yet significant increase in risk among PPI users (RR = 1.15, 95% CI: 1.00-1.32; p = 0.04; I² = 69%). Additional adverse events, including hypomagnesemia and acute kidney injury, were more frequent in PPI users. Asymmetry in the funnel plot suggested publication bias. PPI use is associated with an increased risk of CKD occurrence and ESRD, with a possible but uncertain link to CKD progression. Clinicians should carefully consider long-term PPI therapy, ensure appropriate indications, and monitor renal function in chronic users.

## Introduction and background

Chronic kidney disease (CKD) is a growing global health problem, due to which millions (>800 million) of people are affected worldwide [1]. There is a significant burden on healthcare systems due to the growing burden of CKD. It is also associated with a reduction in quality of life and an increase in hospitalization rate, with a higher risk of cardiovascular complications and mortality [2]. This condition often develops slowly with almost no symptoms in its early stage. Therefore, identifying preventable risk factors is essential to improving long-term outcomes and reducing their public health impact [3].

Proton pump inhibitors (PPIs) are one of the most commonly prescribed medications across the world. They are used widely to treat conditions like gastroesophageal reflux disease (GERD), peptic ulcer disease, and several other acid-related gastrointestinal conditions [4]. Moreover, in both hospital and outpatient settings, the use of PPIs is often preferred because of their strong acid-suppressing effects and very good short-term safety profile. However, their availability over the counter in many countries has also contributed to inappropriate use and long-term side effects [5].

Although PPIs have long been considered safe, several concerns have emerged over the past decade because of the potential adverse effects related to prolonged therapy. These concerns were increased risks of infections, bone fractures, micronutrient deficiencies, and kidney-related complications [6]. Several reports linked PPI use to acute interstitial nephritis (AIN), which was among the earliest signals that suggested that these medications may affect kidney function. AIN can lead to acute kidney injury (AKI), and repeated or unrecognized episodes of AKI are known predictors of CKD development [7].

Due to the growing use of PPIs for extended durations, several observational studies have explored that long-term exposure may be associated not only with acute renal injury but also with chronic and progressive renal decline [8]. Some large cohort studies reported that PPI users may have a higher risk of developing CKD as compared with non-users or with users of the histamine-2 receptor antagonists (H2RAs) [9]. Proposed mechanisms include repeated episodes of subclinical AIN, electrolyte disturbances such as hypomagnesemia, and chronic low-grade inflammation that may contribute to long-term renal damage [10].

However, the evidence linking PPIs to CKD remains inconsistent. Many studies rely on observational designs, making it difficult to establish a causal relationship. Some well-designed studies have not demonstrated a significant association, even after adjusting for confounders, suggesting that the relationship may be more complex than initially thought [11].

Despite these uncertainties, the potential link between long-term PPI use and CKD has raised important clinical concerns. One of the concerns is how often these drugs are prescribed and continued without a clear long-term need. Even a small increase in the CKD risk due to PPI use could have a major public health implication. Thus, there is a need for a systematic review and meta-analysis to clarify this association. It will guide safer prescribing and will support appropriate monitoring for patients who require prolonged acid-suppression therapy.

## Review

This study was conducted as a systematic review and meta-analysis in accordance with the Preferred Reporting Items for Systematic Reviews and Meta-Analyses (PRISMA 2020) guidelines [12].

Inclusion criteria comprised adults aged ≥18 years from any clinical or general population setting; exposure to PPIs, including omeprazole, esomeprazole, pantoprazole, lansoprazole, rabeprazole, or dexlansoprazole; comparison with non-use of PPIs, placebo, or active comparators such as H2RAs; reported outcomes of incident CKD, CKD progression (including estimated glomerular filtration rate decline, doubling of serum creatinine, or progression to a higher CKD stage), or end-stage kidney disease (ESKD/ESRD); primary observational study designs (cohort or case-control); and studies published as full-text, peer-reviewed articles in English.

Exclusion criteria included studies involving only pediatric populations; studies that did not assess PPIs separately or focused exclusively on other acid-suppressive agents; studies without an appropriate comparator group; studies reporting only AKI without CKD outcomes or lacking extractable renal outcome data; reviews, meta-analyses, editorials, letters, case reports, conference abstracts; animal or laboratory studies; and non-English publications. A comprehensive literature search was conducted in the following electronic databases: PubMed/MEDLINE and Google Scholar.

The search covered the last 10 years in the database, from 2015 to 2025. Additionally, reference lists of included articles and relevant prior reviews were manually screened to identify additional eligible studies. No restrictions were placed on geographic location. Only studies published in English were included due to resource limitations.

The search strategy combined controlled vocabulary (e.g., MeSH terms) and free-text keywords related to PPIs and CKD. Boolean operators and database-specific filters were applied. A sample MEDLINE search strategy included the following terms:

(“proton pump inhibitors” OR “omeprazole” OR “lansoprazole” OR “pantoprazole” OR “esomeprazole” OR “rabeprazole” OR “dexlansoprazole”) AND (“chronic kidney disease” OR “CKD” OR “renal insufficiency” OR “kidney failure” OR “end-stage kidney disease” OR “eGFR decline”).

Search strategies for other databases were adapted accordingly. Detailed search strings for databases are provided in the Appendices.

**Figure 1:**
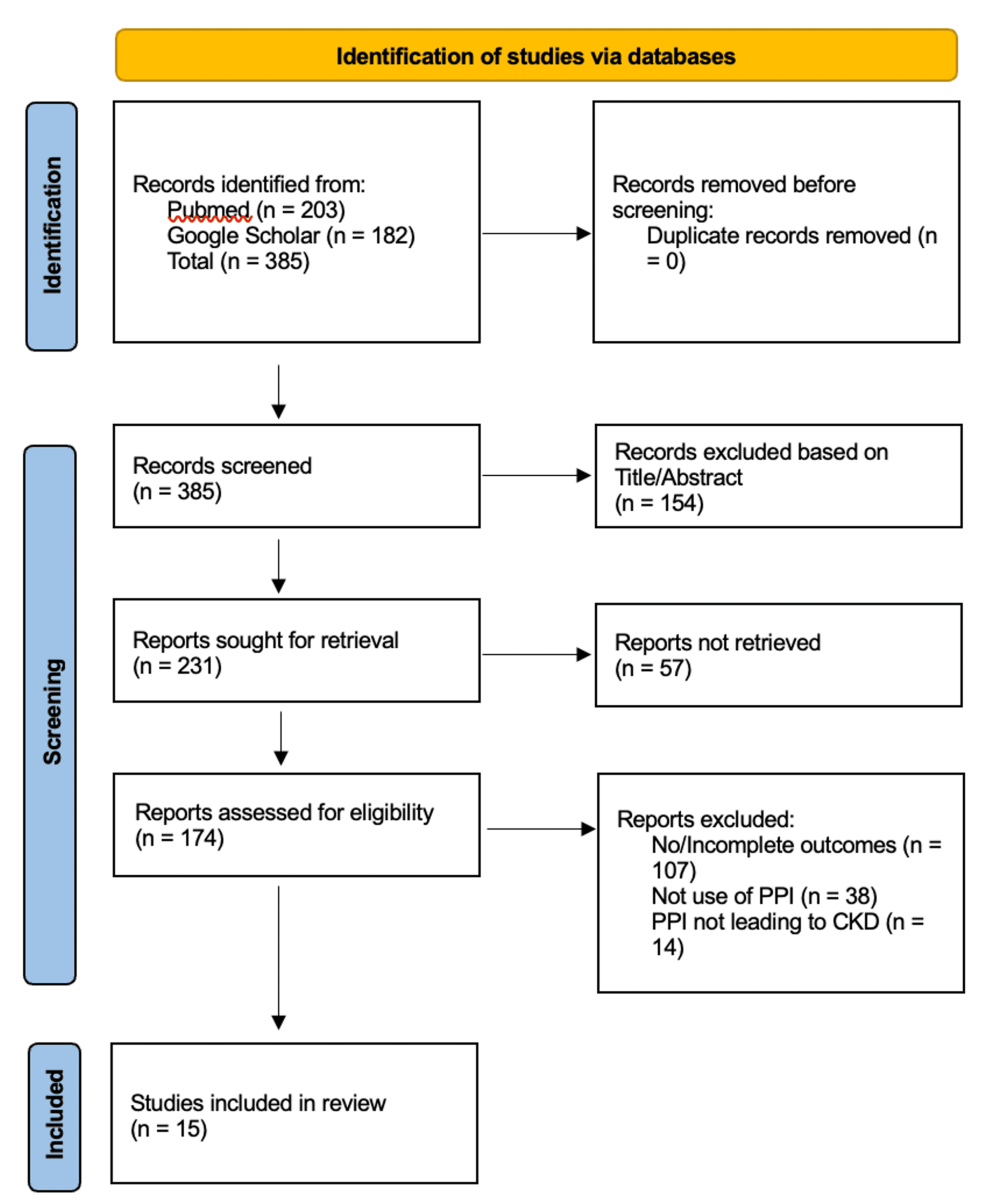
PRISMA chart showing selection and inclusion of studies PRISMA: Preferred Reporting Items for Systematic Reviews and Meta-Analyses [12]

All retrieved records were imported into EndNote software (Clarivate Plc, Philadelphia, PA, USA), and duplicate entries were removed. Two reviewers independently screened titles and abstracts for relevance. Full texts of potentially eligible articles were retrieved and independently assessed by the same reviewers against the predefined inclusion criteria. Differences were resolved by discussion or, if needed, by a third reviewer. The study selection process is summarized visually using the PRISMA 2020 flow chart (Figure 1).

A standardized data extraction form in Microsoft Excel (Microsoft Corp., Redmond, WA, USA) was developed prior to the review. Two reviewers independently extracted data from each included study, with disagreements resolved by consensus. Extracted data mainly include population, intervention, comparison, and outcomes (PICO) from the included studies.

**Figure 2:**
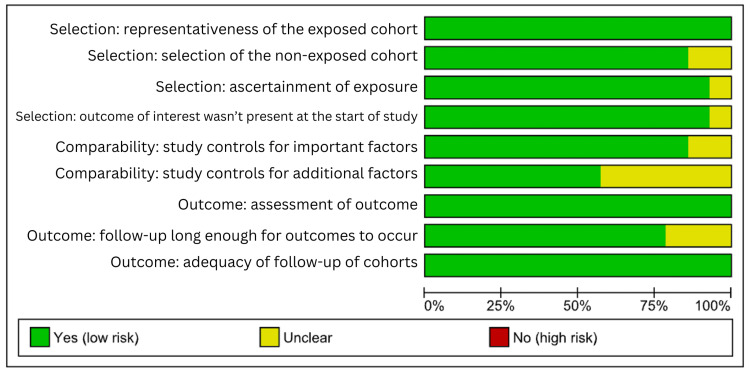
Summary and detail of risk of bias in included studies

**Figure 3:**
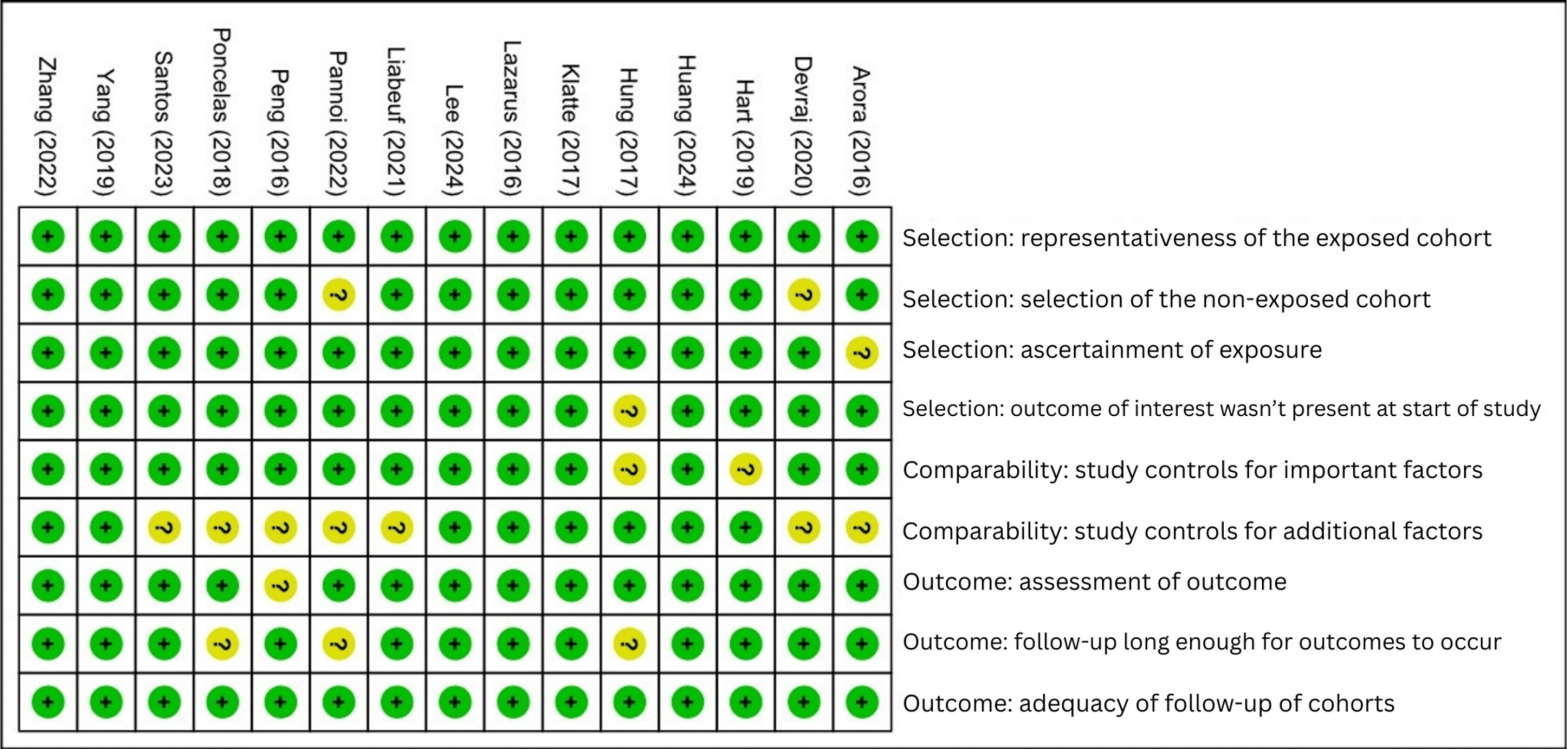
Summary and detail of risk of bias in included studies [14-28]

The risk-of-bias assessment is conducted using the Newcastle-Ottawa Scale, which indicates that most studies demonstrate a low to moderate overall risk of bias across key domains [13]. Figures 2-3 showed that the majority of studies scored well on participant selection and exposure assessment. This reflected the appropriate cohort definitions and reliable documentation of PPI use. However, variability was observed in the comparability domain, where several studies provided limited adjustment for confounding factors, including baseline kidney function, comorbidities, and concurrent medications.

The assessment of outcome was generally adequate, though a few studies showed unclear risk due to insufficient information on follow-up completeness or outcome measurement methods. Several cohorts, including Lee et al. (2024) [14], Huang et al. (2024) [15], and Zhang et al. (2022) [17], showed strong methodological quality with clear participant selection and reliable exposure measurement. Other studies, such as those by dos Santos et al. (2023) [16] and Yang et al. (2019) [21], also performed well but raised minor concerns about residual confounding. A few studies, such as Hung et al. (2017) [25] and Peng et al. (2016) [26], displayed a higher risk in comparability due to limited adjustment for baseline kidney function and comorbidities. Outcome assessment was generally adequate, though some studies (e.g., Devraj and Deshpande, 2020 [20]; Arora et al., 2016 [28]) provided less detail on follow-up completeness.

Results

Our review included 15 studies to assess the prevalence of CKD among PPI users. Notably, the study included large, diverse populations from Asia, Europe, and the United States, with sample sizes ranging from 3,023 to 462,421 participants. Most cohorts consisted of older adults, a group commonly using PPIs. For example, Lee et al. (2024) [14] reported that nearly half of the participants were over 75 years old, while Huang et al. (2024) [15] showed mean ages of 69.7-71.0 years. Several studies also included middle-aged populations, such as dos Santos et al. (2023) [16], with a mean age of ~51.5 years, and Rodríguez-Poncelas et al. (2018) [23], with a mean age of 41.2 years.

Comorbidity burdens were consistently high. In Lee et al. (2024) [14], hypertension affected 91.5%, diabetes 68.3%, and CKD stages 3-4 accounted for 100% of the sample. Huang et al. (2024) [15] also studied advanced CKD (stages 3b-5) with eGFR <45 mL/min/1.73 m². Community-based cohorts showed lower but still relevant comorbidity levels. A study by dos Santos et al. (2023) [16] reported obesity (22%), diabetes (9%), and hypertension (34%). In Rodríguez-Poncelas et al. (2018) [23], baseline eGFR averaged 86.6 mL/min/1.73 m².

The distribution of gender varies across studies, though many cohorts included slightly more males, such as Yang et al. (2019) [21], which reported 59.5% male participants. Follow-up durations ranged from 0.25 years (Pannoi et al., 2022 [18]) to 13.9 years (Lazarus et al., 2016 [27]), providing both short- and long-term perspectives (Table 1).

**Table 1:** Baseline characteristics of included studies assessing the association between PPI use and risk of CKD HTN: hypertension, DM: diabetes mellitus, CHF: congestive heart failure, CVD: cardiovascular disease, IHD: ischemic heart disease, GERD: gastroesophageal reflux disease, CKD: chronic kidney disease, eGFR: estimated glomerular filtration rate, ACR: albumin-to-creatinine ratio, PPI: proton pump inhibitor, H2B: histamine-2 receptor blocker, NR: not reported [14-28]

Author (year)	Country/region	Study design	Study period	Sample size	Follow-up duration	Mean age	Sex distribution	Baseline health status	Baseline kidney function
Lee et al. (2024) [14]	South Korea	Retrospective cohort	2012-2021	34,656	Up to 3 years	<65: 27.3%; 65-74: 28.2%; >75: 44.5%	Male 59.8%; female 40.2%	Diabetes 68.3%; HTN 91.5%; CHF 27.2%	CKD stage 3: 65.1%; stage 4: 34.9%
Huang et al. (2024) [15]	Taiwan	Retrospective cohort	2011-2018	83,432	Up to 1 year	H2B: 69.7 ± 13.3; PPI: 71.0 ± 12.9	H2B male 55.6%; PPI male 61.5%	CKD stage 3b-5	eGFR <45 mL/min/1.73 m²
dos Santos et al. (2023) [16]	Brazil	Prospective cohort	2008-2014	13,301	Mean 3.9 years	~51.5 yrs	Female ~55%; male ~45%	Obesity 22%; diabetes 9%; HTN 34%; CVD 6%	Baseline eGFR: users 84.9; non-users 87.2
Zhang et al. (2022) [17]	UK	Prospective cohort	2006-2017	462,421	Median 8.1 years	Users 59.36; non-users 55.93	Users male 45.6%; non-users 46.4%	Diabetes 12%; hyperlipidemia 69%; CVD 19%; GERD 40%	NR
Pannoi et al. (2022) [18]	Thailand	Retrospective cohort	2010-2017	63,595	Median 0.25 years	Majority ≤60 yrs	PPI: female 63%	HTN 3.9%; DM 0.53%; CVD 0.1%	eGFR ≥90: 57.6% (PPI)
Liabeuf et al. (2021) [19]	France	Prospective cohort	2013-2019	3,023	Median 3.9 years	67 ± 13 yrs	Male 65%; female 35%	CKD stages 2-5	Median eGFR ≈ 32
Devraj and Deshpande (2020) [20]	USA	Retrospective cross-sectional	2009-2013	18,504	NR	Overall 46.3; users 59.8	Male 48%; female 52%	General health fair/poor 17.7%	CKD 3-4 ≈ 4.5%
Yang et al. (2019) [21]	Taiwan	Retrospective cohort	2002-2013	29,970	NR	59.1 ± 11.9	Male 59.5%; female 40.5%	HTN 35.5%; IHD 9.5%; CHF 3.3%	NR
Hart et al. (2019) [22]	USA	Retrospective cohort	1993-2008	84,600	Median 6.8 years	Users 53.4; non-users 42.4	Female 62.8% (users)	Diabetes ~11%; HTN ~27%	NR
Rodríguez-Poncelas et al. (2018) [23]	Spain	Retrospective cohort	2005-2012	51,360	2005-2012	Mean 41.2 yrs	Male 50%; female 50%	Obesity 14%; HTN 15%; diabetes 17%	eGFR mean 86.6
Klatte et al. (2017) [24]	Sweden	Retrospective cohort	2007-2010	114,883	2.7 years	H2B: 55.4; PPI: 62.4	PPI males 39.7%	Diabetes 14%; HTN 50%	eGFR: PPI 88.6
Hung et al. (2017) [25]	Taiwan	Case-control	2000-2013	33,408	~4 months exposure	Cases 64.3 yrs	Male ~59%	HTN 83%; CVD 58%; diabetes 32%	NR
Peng et al. (2016) [26]	Taiwan	Case-control	2006-2011	7,616	~4 years	Cases 65.4 yrs	Male 52%	All had renal disease	NR
Lazarus et al. (2016) [27]	USA	Prospective cohort	1996-2011	10,482	Median 13.9 years	62.8 ± 5.5	Male 42.5%; female 57.5%	HTN 54%; diabetes 15%; CVD 14%	eGFR 87.8; ACR 4 mg/g
Arora et al. (2016) [28]	USA	Retrospective case-control	2001-2008	99,269	Mean 12.4 quarters	~56.6 yrs	Female ~6%	Diabetes 17%; HTN 62%	NR

A wide range of PPIs were evaluated, with omeprazole, esomeprazole, pantoprazole, lansoprazole, and rabeprazole being the most commonly studied agents. Several studies included multiple PPI formulations, such as Lee et al. (2024) [14] and Huang et al. (2024) [15], while others focused mainly on omeprazole, such as Pannoi et al. (2022) [18], in which omeprazole accounted for 95% of prescriptions. Dose reporting was limited, though Liabeuf et al. (2021) [19] provided detailed DDD categories, and Peng et al. (2016) [26] reported dose thresholds using DDD cutoffs. The duration of PPI exposure varied considerably. Lee et al. (2024) [14] defined long-term use as ≥90 days, while dos Santos et al. (2023) [16] evaluated six months of therapy, and Pannoi et al. (2022) [18] reported outcomes after a short median follow-up of 0.25 years. Zhang et al. (2022) [17] assessed regular PPI use based on intake most days of the week. Notably, the comparator groups also differed across studies. Many used H2RA users as the reference group, such as Lee et al. (2024) [14], Huang et al. (2024) [15], and Klatte et al. (2017) [24], while others compared PPI users with non-users (dos Santos et al., 2023 [16]; Rodríguez-Poncelas et al., 2018 [23]). Comparator health profiles often reflected lower comorbidity levels and better baseline kidney function; for example, baseline eGFR was 87.2 mL/min/1.73 m² among non-users in dos Santos et al. (2023) (Table 2) [16].

**Table 2:** Characteristics of PPI exposure and comparator groups across included studies evaluating the association between PPI use and CKD PPI: proton pump inhibitor, H2RA: histamine-2 receptor antagonist, DDD: defined daily dose, CKD: chronic kidney disease, HTN: hypertension, CHF: congestive heart failure, CVD: cardiovascular disease, GERD: gastroesophageal reflux disease, GI: gastrointestinal, eGFR: estimated glomerular filtration rate, NR: not reported, HR: hazard ratio, ORs: odds ratios [14-28]

Author (year)	PPI type	PPI dose	Duration of PPI use	Comparator type	Comparator health and kidney status
Lee et al. (2024) [14]	Omeprazole, esomeprazole, pantoprazole, lansoprazole, rabeprazole, dexlansoprazole	NR	≥90 days continuous use	H2RA users	CKD stage 4: 34.5%; diabetes: 66.7%; HTN: 89.8%; CHF: 23.9%
Huang et al. (2024) [15]	Dexlansoprazole, omeprazole, esomeprazole, lansoprazole, pantoprazole, rabeprazole	NR	NR	H2RA users	GERD 8.6%; GI bleed 3.6%; peptic ulcer 15.5%; CVD 72.9%; HTN 82.6%; diabetes 56%; eGFR NR
dos Santos et al. (2023) [16]	Omeprazole, esomeprazole, lansoprazole, pantoprazole, rabeprazole	NR	6 months	Non-users	Lower comorbidity; eGFR 87.2 ± 13.5
Zhang et al. (2022) [17]	Omeprazole, lansoprazole, pantoprazole, rabeprazole, esomeprazole	NR	Most days of the week in the last 4 weeks	Non-users; H2RA users	H2RA users: HR 1.10 (0.96-1.25) weighted
Pannoi et al. (2022) [18]	Omeprazole (95%)	NR	90-day grace period; follow-up 0.25 years	H2RA	eGFR ≥90: 63.3%; <90: 36.7%
Liabeuf et al. (2021) [19]	Omeprazole, pantoprazole, esomeprazole, lansoprazole, rabeprazole	DDD ≤30 mg/day (n = 678); ≥40 mg/day (n = 283)	New users: median 1 year	Non-users	Baseline eGFR 32.2 (23.2-41.9)
Devraj and Deshpande (2020) [20]	Omeprazole, esomeprazole, pantoprazole, lansoprazole, dexlansoprazole, rabeprazole	NR	NR	(1) No medication, (2) non-PPI meds	No-med: CKD1 40.5%; non-PPI: CKD1 36.4%
Yang et al. (2019) [21]	Esomeprazole, lansoprazole, omeprazole, pantoprazole, rabeprazole	>180 DDD	>180 DDD within 1 year	No PPI exposure	No CKD at baseline
Hart et al. (2019) [22]	Esomeprazole, lansoprazole, omeprazole, pantoprazole, rabeprazole	NR	NR	Non-users; H2RA users	NR
Rodríguez-Poncelas et al. (2018) [23]	Omeprazole, esomeprazole, lansoprazole, pantoprazole, rabeprazole	Standard vs high dose	<1 mo; 1-3 mo; 3-6 mo; 6-12 mo; 12-24 mo; >24 mo	Non-users	Baseline eGFR 86.60; obesity 10%; HTN 10%; diabetes 10%
Klatte et al. (2017) [24]	Omeprazole, pantoprazole, lansoprazole, esomeprazole, rabeprazole	NR	NR	H2RA (mostly ranitidine)	eGFR 94.2; HTN 38.8%; diabetes 11.6%
Hung et al. (2017) [25]	Esomeprazole, lansoprazole, omeprazole, pantoprazole, rabeprazole	ORs reported per PPI	Effect per month; mean ~4 months	Non-users; H2RA users	NR
Peng et al. (2016) [26]	Omeprazole, pantoprazole, lansoprazole, rabeprazole, esomeprazole	DDD cutoffs reported	NR	Non-use of PPI and H2RA	NR
Lazarus et al. (2016) [27]	NR	NR	NR	Non-users; H2RA users	H2RA eGFR 86.5 ± 13.5
Arora et al. (2016) [28]	NR	NR	NR	No PPI exposure	Comorbidity data reported; kidney status NR

All the studies consistently reported a higher risk of kidney-related outcomes among PPI users compared with non-users or H2RA users. Several large cohort studies have demonstrated an increased incidence of CKD among PPI users. For example, Huang et al. (2024) [15] found a CKD incidence of 9.71% in PPI users vs 7.36% in H2RA users, while Hart et al. (2019) [22] reported a CKD incidence of 15.3% in PPI users compared with 6.42% in non-users. Similarly, Zhang et al. (2022) [17] observed higher CKD incidence rates among PPI users (4.45 per 1,000 person-years) relative to non-users (1.63 per 1,000 person-years). Evidence for CKD progression also points toward an elevated risk with PPI exposure. Lee et al. (2024) [14] reported an incidence rate ratio of 1.12 for progression events among PPI users vs H2RA users, while Huang et al. (2024) [15] showed an adjusted hazard ratio of 1.495 for progression to ESRD. Peng et al. (2016) [26] further demonstrated that 50% of participants reached ESRD during follow-up. Some studies also reported additional adverse effects. Pannoi et al. (2022) [18] found markedly higher rates of hypomagnesemia among PPI users (310 cases) than among H2RA users (16 cases), and Zhang et al. (2022) [17] reported increased risks of infections, fractures, and diabetes. Mortality reporting was limited, though Arora et al. (2016) [28] documented an all-cause mortality rate of 11.84% (Table 3).

**Table 3:** Renal and clinical outcomes reported across included studies evaluating the association between PPI use and CKD CKD: chronic kidney disease, ESKD: end-stage kidney disease, ESRD: end-stage renal disease, eGFR: estimated glomerular filtration rate, PPI: proton pump inhibitor, H2RA: histamine-2 receptor antagonist, IR: incidence rate, IRR: incidence rate ratio, HR: hazard ratio, Adj HR: adjusted hazard ratio, PSM: propensity score matching, AKI: acute kidney injury, AIN: acute interstitial nephritis, NR: not reported [14-28]

Author (year)	CKD incidence	CKD progression	ESKD/ESRD cases	Other adverse effects	Mortality (N, %)
Lee et al. (2024) [14]	NR	PPI: IR 10.21/100 PYs; H2RA: 9.16/100 PYs; Crude IRR 1.12 (1.05-1.19)	Event counts: PPI 1,113; H2RA 1,214 (PSM)	NR	NR
Huang et al. (2024) [15]	PPI: 9.71% vs H2RA: 7.36%	Adj HR 1.495 (1.198-1.867)	PPI 102; H2RA 378	AKI: PPI 6.18% vs H2RA 4.81%	NR
dos Santos et al. (2023) [16]	Total CKD 3.8%; non-users 3.6%; PPI users 6.5%	eGFR decline is greater in PPI users	NR	NR	NR
Zhang et al. (2022) [17]	PPI: 4.45/1000 py; non-users: 1.63/1000 py	NR	NR	Hypomagnesemia, infections, fractures, diabetes	NR
Pannoi et al. (2022) [18]	PPI: 3.21/100 PY; H2RA: 1.11/100 PY (before PSM)	NR	NR	Hypomagnesemia: PPI 310 vs H2RA 16; AKI: 1 PPI	NR
Liabeuf et al. (2021) [19]	NR	NR	526 (overall); 354 in non-user subgroup	GI symptoms (diarrhea, pain, constipation), headache	374/2,900 (12.9%); Subgroup 216/1,940
Devraj and Deshpande (2020) [20]	NR	NR	NR	NR	NR
Yang et al. (2019) [21]	No-PPI 12.1%; PPI 12.6%	NR	NR	Anemia 0.9% in the exposure group	NR
Hart et al. (2019) [22]	PPI: 15.3% vs non-users: 6.42%	NR	NR	NR	NR
Rodríguez-Poncelas et al. (2018) [23]	7-48% depending on duration/exposure category	NR	NR	NR	NR
Klatte et al. (2017) [24]	H2RA: 3.94/1000 py	30% eGFR decline, HR 1.26; creatinine doubling, HR 1.26	H2RA 3; PPI 128	Fractures HR 1.21; hyponatremia HR 1.15	NR
Hung et al. (2017) [25]	16,704 CKD cases (overall)	NR	NR	NR	NR
Peng et al. (2016) [26]	NR	Progression endpoint = ESRD	3,808/7,616 = 50%	Hypomagnesemia, AIN noted	NR
Lazarus et al. (2016) [27]	PPI: 14.2/1000 py; non-users: 10.7/1000 py	NR	NR	AKI events reported	NR
Arora et al. (2016) [28]	19,311/76,462 developed CKD	NR	NR	NR	11,758 (11.84%)

Meta-analysis for the quantitative evidence of the association of PPI with CKD

**Figure 4:**
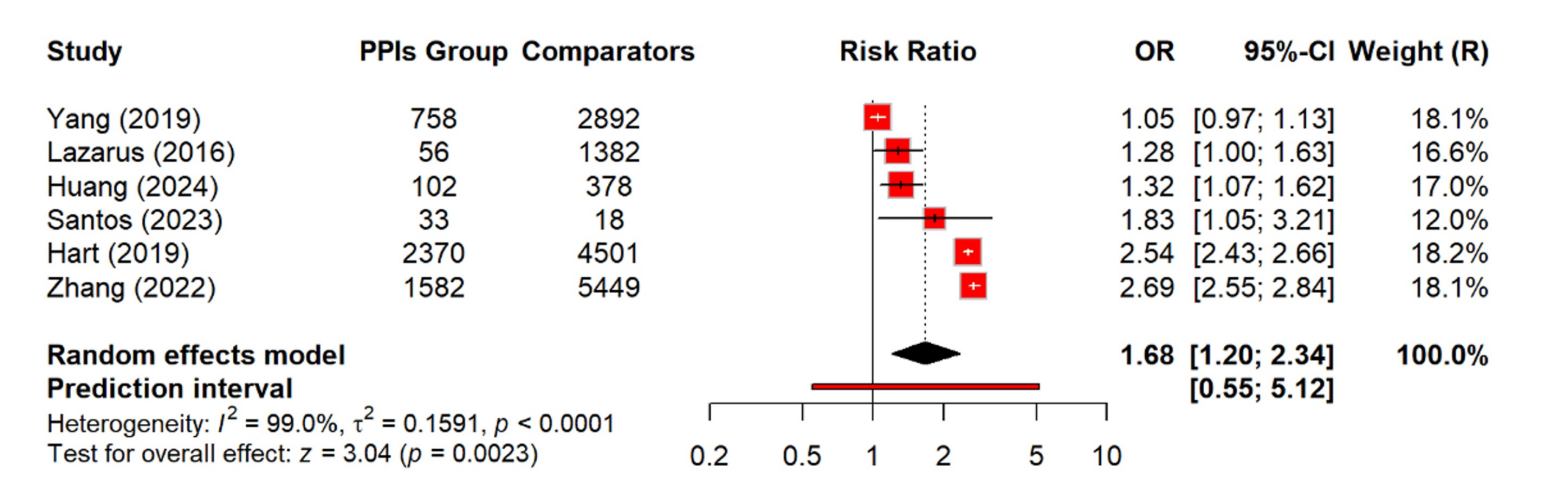
Risk of occurrence of CKD among PPI users OR: odds ratio, CI: confidence interval, CKD: chronic kidney disease, PPI: proton pump inhibitor [14-16,20,21,26]

A pooled analysis of 6 studies involving 594,680 participants showed a significantly higher risk of developing CKD among PPI users. The random-effects model estimates a relative risk of 1.68 (95% CI: 1.20-2.34; p = 0.002). This indicated a meaningful association between PPI exposure and CKD. The heterogeneity across studies was extremely high (I² = 99%), which reflected substantial variability in effect estimates (Figure 4).

**Figure 5:**
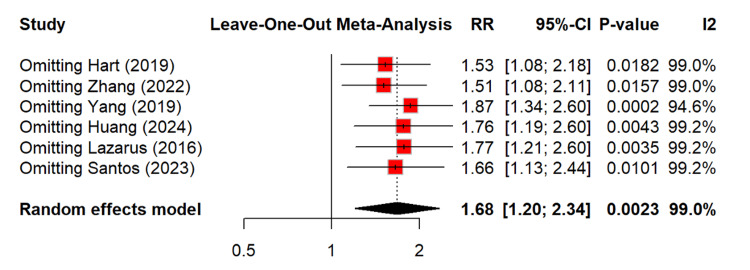
Leave-one-out analysis for occurrence of CKD among PPI users RR: relative risk, CI: confidence interval, CKD: chronic kidney disease, PPI: proton pump inhibitor [14-16,20,21,26]

To identify the source of heterogeneity, we performed a leave-one-out meta-analysis, which showed that none of the study removals affected overall heterogeneity (Figure 5).

**Figure 6:**
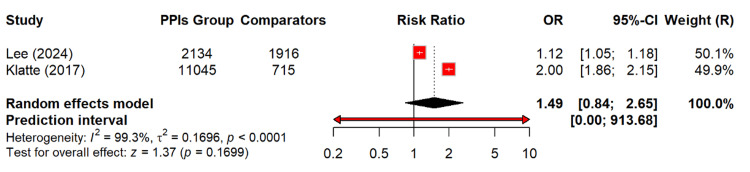
Risk of CKD progression among PPI users OR: odds ratio, CI: confidence interval, CKD: chronic kidney disease, PPI: proton pump inhibitor [13,23]

Figure 6 shows that PPI use among 171,583 patients is associated with a higher risk of CKD progression, but this difference is not significant. The relative risk of this effect is 1.49 (95% CI: 0.84-2.65; p = 0.17). This indicated that the true effect may range from no increased risk to a possible substantial increase. The heterogeneity of this effect size was extremely high (I² = 99.3%).

**Figure 7:**
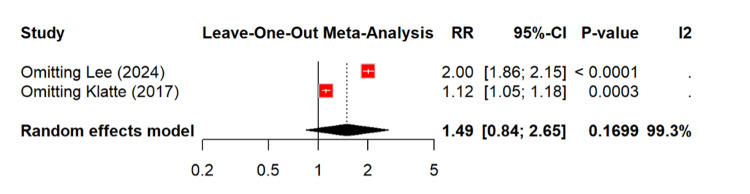
Leave-one-out analysis for CKD progression among PPI users RR: relative risk, CI: confidence interval, CKD: chronic kidney diseas, PPI: proton pump inhibitor [13,23]

Figure 7 shows that leave-one-out analysis wasn’t able to identify the source of heterogeneity.

**Figure 8:**
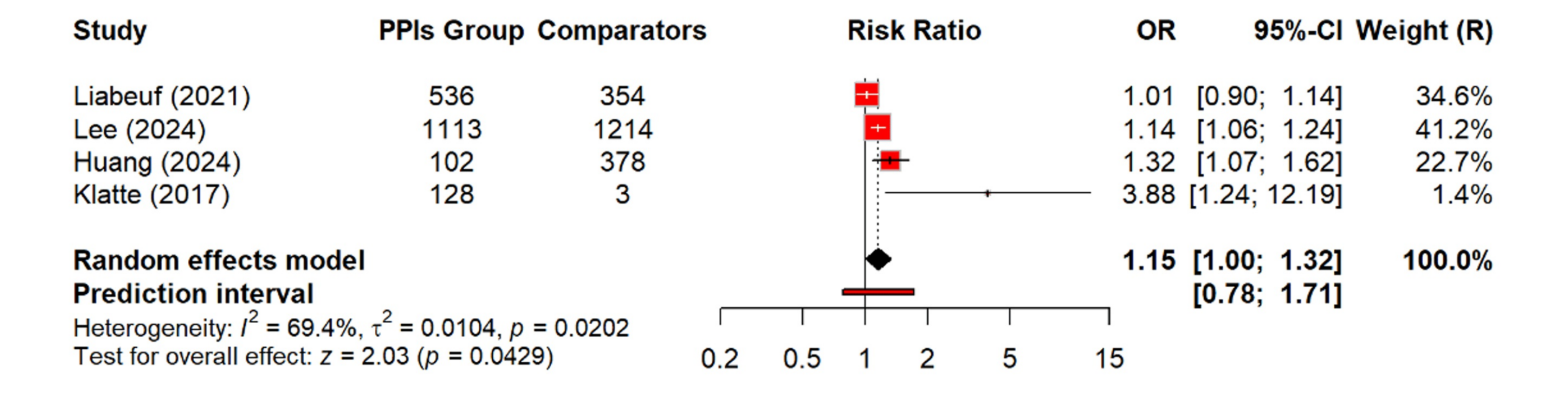
Risk of ESRD among PPI users OR: odds ratio, CI: confidence interval, ESRD: end-stage renal disease, PPI: proton pump inhibitor [13-14,18,23]

Figure 8 shows a modest but statistically significant increase in the risk of ESRD among PPI users. The random-effects model yields a relative risk of 1.15 (95% CI: 1.00-1.32; p = 0.04). This suggests that PPI exposure may be associated with the small increase in ESRD risk. The heterogeneity was moderate (I² = 69%), indicating some variability between studies but not enough to obscure the overall effect.

**Figure 9:**
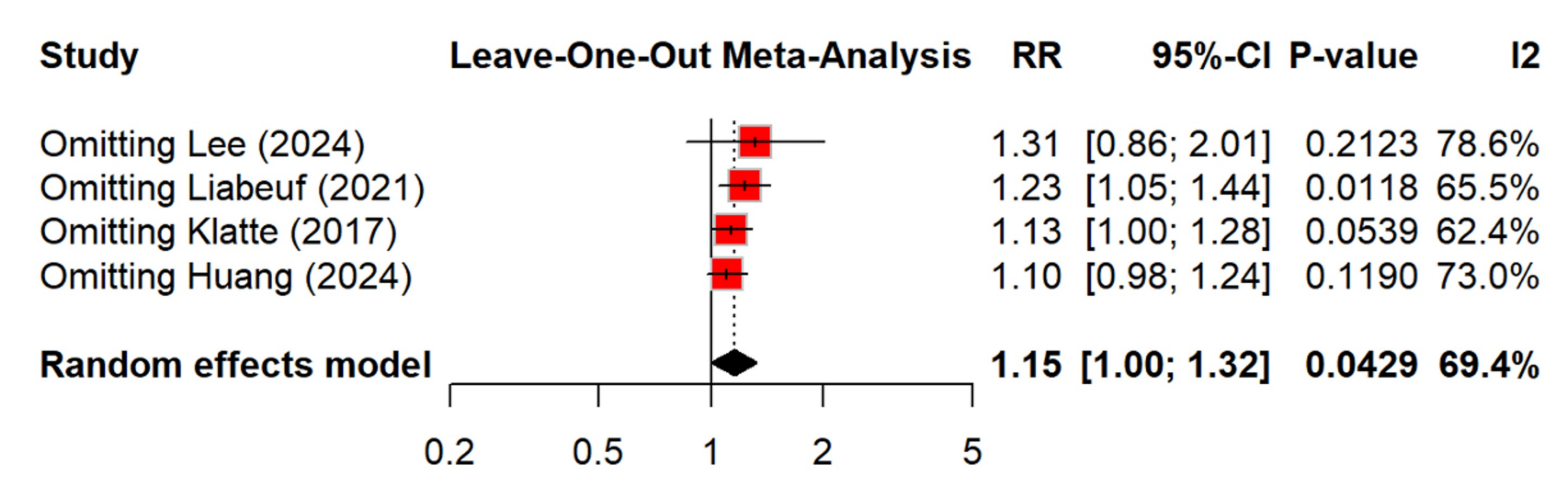
Leave-one-out analysis for risk of ESRD among PPI users RR: relative risk, CI: confidence interval, ESRD: end-stage renal disease, PPI: proton pump inhibitor [13-14,18,23]

Figure 9 shows that leave-one-out analysis did not identify the source of heterogeneity.

**Figure 10:**
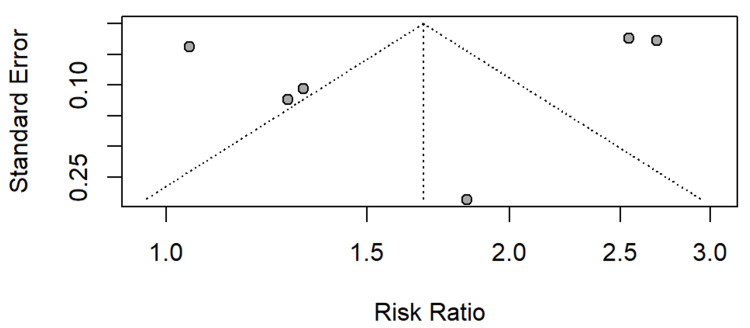
Assessment of publication bias

Figure 10 shows the funnel plot, which reveals an asymmetric distribution of study effect sizes, suggesting publication bias. Several studies lie outside the expected triangular region, with smaller studies tending to report larger risk estimates. This pattern indicates that smaller negative or null studies may be missing from the literature. The imbalance around the pooled effect also supports the possibility of selective publication or reporting.

Discussion

CKD is a major global health challenge that is linked to poorer quality of life, higher hospitalization rates, and increased risk of cardiovascular events [29]. Because this condition often develops silently, understanding the preventable risk factors is crucial. The medications like PPIs are widely used for acid-related conditions and are frequently continued long-term, sometimes without medical necessity. Although these are generally considered safe, several concerns have emerged about potential kidney-related harm, which includes interstitial nephritis and chronic renal decline [30]. Due to unclear or mixed evidence, a systematic review and meta-analysis are needed to clarify the association and guide safer, more informed prescribing.

Notably, our review found a robust association between PPI use and incident CKD. A substantial body of prior research strengthens these findings. For instance, the landmark study by Edinoff et al. (2023) demonstrated that PPI users had a significantly higher risk of incident CKD than non-users, even after accounting for confounding factors [31]. Similarly, Awdishu and Abagyan (2022) showed that the risk of ESKD or >50% decline in eGFR was elevated in patients treated with PPIs (HR = 1.47; 95% CI, 1.38 to 1.57) [32]. This suggested that the association is not merely due to acid-suppressive therapy but may be specific to PPIs.

Several mechanisms support this relationship. PPIs are among the most commonly implicated drugs in AIN, described in multiple case reports and series (Blank et al., 2014) [33]. Although AIN may initially present subtly or remain unrecognized, persistent inflammation can lead to chronic tubulointerstitial fibrosis, a pathway strongly associated with progressive CKD. Chronic hypomagnesemia, another established adverse effect of PPIs, may also contribute to renal dysfunction through tubular injury, which is aligned with a study by Gommers et al. (2022) [34].

Moreover, our findings suggested a trend toward increased CKD progression among PPI users, although the effect was not statistically significant. This aligns with earlier studies showing that PPI use may accelerate renal deterioration. A study by Sharma et al. (2025) reported a faster decline in eGFR among long-term PPI users compared with H2RA users [35]. Other large cohorts, including Xie et al. (2017), also demonstrated higher risks of ≥30% eGFR decline associated with chronic PPI exposure [36].

Notably, the inconsistency in progression outcomes across studies likely reflects methodological variability. The definitions of CKD progression differ widely in the literature, ranging from absolute changes in eGFR to time-to-ESRD outcomes. Moreover, confounding by the indication is another challenge, as patients with multiple comorbidities or polypharmacy are more likely to be prescribed PPIs and may inherently be at higher risk of renal decline. Nonetheless, consistent observations of greater eGFR decline across multiple independent datasets support continued surveillance of renal function in chronic PPI users.

Our meta-analysis also demonstrated a small but statistically significant increase in ESRD risk among PPI users. While the effect size was modest, it is clinically important given the irreversible nature of ESRD. These results complement those of Ang et al. (2024), who found that PPI use was associated with a higher (OR of 1.25) long-term risk of ESRD compared with H2RA users [37]. Another study by Mir et al. (2024) similarly reported that long-term PPI therapy was associated with a significantly increased risk of progression to ESRD, especially in older adults and those with diabetes [38].

Beyond the statistical associations, the clinical context is essential. PPIs are often continued without re-evaluation, sometimes for years after the initial indication has resolved. Previous studies show that 25-70% of PPI prescriptions lack a clear ongoing indication (Forgacs and Loganayagam, 2008) [39]. This overuse magnifies exposure to potential long-term adverse effects, including renal harm.

Further, PPIs may interact with the kidney physiology through multiple mechanisms beyond AIN. Experimental research suggests that PPIs may alter lysosomal acidification, disrupt mitochondrial function, and impair proton pumps in renal tubules, and that these effects could contribute to renal injury [40]. The emerging evidence also links PPI use to alterations in the gut microbiome, which may, in turn, indirectly influence systemic inflammation and renal function.

The substantial heterogeneity observed across studies, which may be due to differences in PPI exposure definitions, population characteristics, comorbidities, and follow-up lengths, all contribute to variability. The funnel plot asymmetry in our study suggested possible publication bias, similar to patterns reported in other PPI safety reviews. It highlighted the need for more transparent reporting of null or negative results.

Clinical implications

Due to the widespread use of PPIs globally and the increasing evidence of the renal risks, clinicians should adopt a more judicious approach to long-term prescribing. The American Gastroenterological Association guidelines recommend regular reassessment, deprescribing when appropriate, and considering alternatives, such as H2RAs. These findings should reinforce these recommendations, especially for older adults and individuals with pre-existing renal impairment.

## Conclusions

Our review supports the growing body of evidence that shows long-term PPI use may increase the risk of CKD, ESRD, and other renal complications. While causality cannot be definitively established, the consistency of findings across multiple large observational studies and plausible biological mechanisms warrants caution. Clinicians should regularly re-evaluate PPI therapy, ensure appropriate indications, and consider renal monitoring in chronic users to mitigate potential risks.

## Appendices

**Table 4:** Detailed search strategy for each database

Database	Platform/ interface	Search string
PubMed (MEDLINE)	NCBI	(("Proton Pump Inhibitors"[Mesh] OR "proton pump inhibitor*" OR PPI OR PPIs OR omeprazole OR esomeprazole OR pantoprazole OR rabeprazole OR lansoprazole OR dexlansoprazole)) AND (("Chronic Kidney Disease"[Mesh] OR "Kidney Failure, Chronic"[Mesh] OR "chronic kidney disease" OR CKD OR "end-stage kidney" OR ESKD OR ESRD OR "eGFR decline" OR "renal insufficiency")) AND (observational OR cohort OR "case-control" OR "nested case-control" OR epidemiologic* OR "population-based" OR "longitudinal" OR "prospective" OR "retrospective") NOT (review[pt] OR systematic [12] OR meta-analysis[pt] OR editorial[pt] OR letter[pt] OR comment[pt] OR case reports[pt])
Google Scholar	Google Scholar Web Interface	"proton pump inhibitor" "chronic kidney disease" cohort -review -meta -systematic -protocol
